# Quercetin Relieves the Excised Great Saphenous Vein Oxidative Damage and Inflammatory Reaction

**DOI:** 10.1155/2021/6251559

**Published:** 2021-12-31

**Authors:** Yunpeng Bai, Qingliang Chen, Xiaolong Zhu, Nan Jiang, Ximing Li, Zhigang Guo

**Affiliations:** ^1^Chest Hospital, Tianjin University, Tianjin 300222, China; ^2^Tianjin Chest Hospital, Tianjin Medical University, Tianjin 300222, China

## Abstract

**Objective:**

The patency and quality of transplanted great saphenous vein (GSV) can seriously influence the physical state and life quality of patients who accepted the coronary artery bypass grafting (CABG). Quercetin is known for antioxidant, antithrombotic, anti-inflammatory, and antitumor properties. In this study, we examined the protection of quercetin to the great saphenous vein from oxidative and inflammatory damage.

**Methods:**

The GSVs were collected from 15 patients undergoing CABG and cultured. Treated the veins by H_2_O_2_ and detected the NO, SOD, and MDA content by the relevant kits to explore the quercetin protection against oxidative damage. Then, for another group of GSVs, sheared them and detected the inflammatory cytokines, such as IL-6, TNF*α*, CCL20, PCNA, and VEGF. Collect the veins for H&E staining and PCNA and VEGF immunofluorescent staining.

**Results:**

Pretreatment by quercetin reduced the production of NO and MDA induced by H_2_O_2_, and increased SOD activity. Quercetin also supressed the mRNA expressions of IL-6, TNF*α* after mechanical damage and had no influence on CCL20 and VEGF. Consistent with the lower expression of PCNA treated by quercetin, the vein intima was thinner.

**Conclusion:**

These results demonstrated that quercetin protects GSVs by reducing the oxidative damage and inflammatory response and also suppresses the abnormal thickening of venous endothelium by inhibiting cell proliferation. It reminded that, to some extent, quercetin has the potential to release the great saphenous vein graft damage.

## 1. Introduction 

Quercetin is a kind of flavonoid, which is widely distributed in a variety of fruits, vegetables, and traditional Chinese medicine, such as apple, onion, mulberry parasitism, chrysanthemum, ginkgo biloba, notoginseng, and so on. Quercetin has many beneficial effects on the human body, including antithrombotic, anti-inflammatory, and antitumor properties [1]. It has been regarded as a good antioxidant that removes reactive oxygen species produced naturally in the body, such as O_2_- and ONOO-, and promotes the transfer of zinc into cells as an intracellular antioxidant [2, 3], enhancing antioxidation ability of cells.

The incidence of cardiovascular disease has increased dramatically year by year, becoming a serious health challenges not only in developed countries but also in developing countries, with the improvement and changes of food. Coronary atherosclerosis is a common clinical cardiovascular disease, which is bound up with inflammatory response. Epidemiological research studies have showed that the addition of flavonoids can reduce the risk of cardiovascular disease [4]. Ovsepyan found that macrophages secreted large amounts of C-reactive protein (CRP) and TNF*α*, IL-6, and other proinflammatory factors, promoting the apoptosis of smooth muscle cell and the development of atherosclerosis [5]. Coronary artery bypass surgery (CABG) has become a common cardiac surgery treatment for patients with coronary heart disease (CHD). The migratory vessels are mainly supplied by the patient's own great saphenous vein (GSV), internal mammary artery, or radial artery. The existing clinical studies have showed that patency rate of artery bridge is significantly higher than that of vein bridge after 5 years or 10 years [6]. Gooch believed that the low patency rate of venous bridge is caused by mechanical damage during operation [7]. Research studies also showed that vascular damage is closely related to oxidative stress [8], especially vascular endothelial damage, which enhances the occurrence and development of arteriosclerosis.

Then, reduced oxidative and mechanical damage are beneficial for the GSV patency. Barros put forward that the preservation methods of GSV can affect the vascular patency rate and failure of venous bridge [9]. After the removal of GSV, the blood supply in vitro was insufficient, and the rapid loss of water caused venous spasm or endothelial injury. Studies revealed that improper preservation of GSV could destroy the membrane integrity in vitro [10]. Therefore, many researchers tried to find a proper preservation solution to reduce the injury and improve the GSV quality. Harscamp proposed that buffer salt solution showed superior benefits on GSV than saline solution and whole blood [11].

Then, can we apply some reagents to better protect the GSV? Above all, we know that the excised GSV injury mainly comes from the intraoperative mechanical damage and oxidative damage caused by hypoperfusion. Quercetin plays a good antioxidant and anti-inflammatory role [12]. In this study, we separately designed the oxidative and mechanical damage models in vitro to explore the protective effect of quercetin on the excised GSV.

## 2. Materials and Methods

### 2.1. Patients and GSV Culture

The GSVs were collected from the CABG patients who signed the informed consent. Cleared the perivascular adipose under aseptic conditions, and then, GSV was cut into vascular rings about 3 mm long, divided into 3 groups, and placed in the DMEM medium (Thermo Fisher Scientific, America) containing 20% FBS (Thermo Fisher Scientific, USA) and double antibody (Thermo Fisher Scientific, USA) at 5% CO_2_ and 37°C. The excised GSV culture methods referred to Prasongsukarn [13].

This study has been approved by the Institutional Ethical Committee of Tianjin Chest Hospital.

### 2.2. Oxidative and Mechanical Damage Protocol

The vascular rings were divided into 3 groups: control group, H_2_O_2_ group cultured in DMEM, and H_2_O_2_ + quercetin group cultured in DMEM containing 200 umol/L quercetin (Abcam, UK). After 1 hour, the H_2_O_2_ group and H_2_O_2_ + quercetin group were treated with 100 umol/L H_2_O_2_. After 4 hours, the culture supernatant was recovered, and then, the NO content was detected by the nitric oxide (NO) kit (Solarbio, China). Another 3 groups, treated as above, after 24 h, were treated by 100 umol/L H_2_O_2_. Then, NO, SOD, and MDA contents were detected using the relevant kits (Solarbio, China). In the mechanical damage experiment, shear the veins by scissors to form a 2 mm incised wound. The control group did not do any treatment. The wound group was cultured in DMEM, and the wound + quercetin group was cultured in DMEM containing 200 umol/L quercetin. After 24 hours, the veins were collected, and mRMA levels of IL-6, TNF*α*, CCL20, PCNA, and VEGF were detected by qRT-PCR. Then, the veins were collected for H&E staining, PCNA, and VEGF immunofluorescent staining.

### 2.3. H&E and Immunofluorescence Staining

The paraffin sections were heated in an oven. After hydration, they were treated with eosin staining for 1 minute and hematoxylin for 30 seconds. As for immunofluorescence staining, the hydrated sections were first treated by citrate-based antigen unmasking solutions (Vector Laboratory, America). They were incubated with antibodies (1 : 100) against PCNA (Solarbio, China) and VEGF (Solarbio, China), respectively. The sections were then incubated with commercial secondary antibody (1 : 200). The sections were photographed with Nikon microscopy (Nikon, Japan).

### 2.4. mRNA Analyses

The vein tissues were homogenized with Stat 60 RNA extraction reagent (Tel-Test, TX) with a homogenizer (Kinematica, Switzerland), and then, RNA was extracted and further purified by a RNA easy mini kit (Qiagen, China) for qPCR following the manufacturer's protocol.

The purified RNA was reverse transcribed, and gene expression was evaluated by real-time quantitative PCR. Briefly, cDNA was synthesized, and qPCR was performed using the Power SYBR Green (Applied Biosystems, USA) and 7300 Real-Time PCR system (Applied Biosystems, USA). Reverse transcription was performed following the temperature protocol: 37°C for 1 h and 94°C for 5 min. All primers used are given in Table S1. Subsequently, qPCR was performed using the SYBR Green ER PCR kit (Thermo Fisher Scientific, Inc.). The following cycling conditions were as follows: 95°C for 20 s, followed by 40 cycles of 95°C for 15 sec and 60°C for 1 min. Relative mRNA levels were calculated by comparing with the housekeeping gene GapDH. Quantitative values were calculated using the 2^−ΔΔ*Cq*^ method.

## 3. Results and Discussion

### 3.1. Results

#### 3.1.1. Short-Time Quercetin Pretreatment Did Not Protect the GSV from H_2_O_2_-Induced Damage

**Figure 1:**
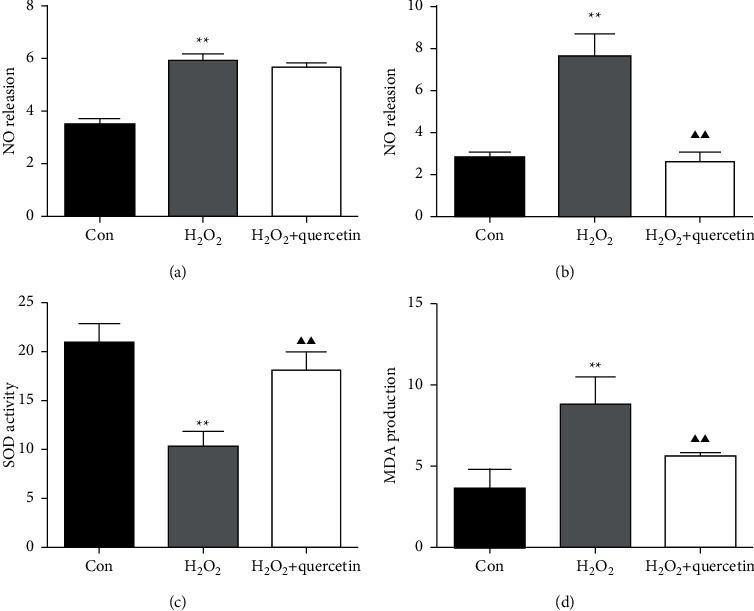
Quercetin pretreatment protects GSV from H_2_O_2_-induced damage. (a) The NO concentration in the culture supernatant, pretreated by quercetin for 1 h. (b) The NO concentration in the culture supernatant, pretreated by quercetin for 24 h. (c) The MDA concentration in the cultured GSV, pretreated by quercetin for 24 h. (d) The SOD concentration in the cultured GSV, pretreated by quercetin for 24 h. Statistical significance compared with H_2_O_2_ GSV vs. Con GSV is shown by an asterisk ^*∗*^*P* < 0.05, ^*∗∗*^*P* < 0.01) and with H_2_O_2_ GSV vs. H_2_O_2_ + quercetin GSV is shown by a trilateral (^*▲*^*P* < 0.05, ^*▲▲*^*P* < 0.01). Mean ± SD shown (*n* = 6).

The GSVs cultured in vitro were induced oxidative damage by 100 umol/L H_2_O_2_. After treated for about 1 hour, the NO generation was detected using the NO kit. After H_2_O_2_-induced damage, the generation of NO increased (*P* < 0.05), revealing that H_2_O_2_ induced oxidative damage. There were no significant statistical differences in NO concentration between H_2_O_2_ and H_2_O_2_ + quercetin groups (*P* > 0.05) (Figure 1(a)).

#### 3.1.2. Long-Time Quercetin Pretreatment Protected the GSV from H_2_O_2_-Induced Damage

We speculated that the protective effect of quercetin might be related to the pretreatment time. Then, we extended the pretreatment to 24 hours and repeated the above experiments; the results revealed the production of oxidative damage decreased. Compared with the H_2_O_2_ group, NO generation was remarkably decreased in the H_2_O_2_ + quercetin group (*P* < 0.01), even lower than that in the control group (Figure 1(b)). We further tested MDA production and SOD activity (Figures 1(c)-1(d)), and found that MDA content in the H_2_O_2_ + quercetin group was lower than that in the H_2_O_2_ group (*P* < 0.01). Furthermore, SOD activity in the H_2_O_2_ + quercetin group was obviously higher than that in the H_2_O_2_ group (*P* < 0.01) and lower than the control group (*P* < 0.05).

#### 3.1.3. Quercetin Suppressed the Inflammatory Responses Induced by Mechanical Damage

**Figure 2:**
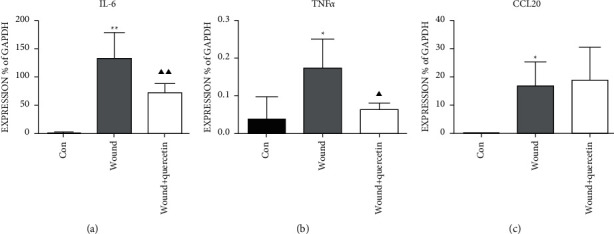
Quercetin pretreatment suppresses the inflammatory responses induced by mechanical damage. (a) The mRNA levels for IL-6 in the 3 groups of cultured GSV, pretreated by quercetin for 24 h. (b) The mRNA levels for TNF*α* in the 3 groups of cultured GSV, which were pretreated by quercetin for 24 h. (c) The mRNA levels for CCL20 in the 3 groups of cultured GSV, pretreated by quercetin for 24 h. Statistical significance compared with H_2_O_2_ GSV vs. Con GSV is shown by an asterisk (^*∗*^*P* < 0.05, ^*∗∗*^*P* < 0.01) and with H_2_O_2_ GSV vs. H_2_O_2_ + quercetin GSV is shown by a trilateral (^*▲*^*P* < 0.05, ^*▲▲*^*P* < 0.01). Mean ± SD shown (*n* = 6).

In order to explore the role of quercetin in the inflammatory response induced by mechanical damage, we established mechanical damage GSV models using a scissor. Then, the mRNA levels of inflammatory cytokines were detected. After quercetin treatment, the transcription levels of IL-6 and TNF*α* reduced (*P* < 0.05), but still higher than the control group (*P* < 0.01, Figures 2(a)-2(b)). However, the expression of CCL20 showed no significant relation to quercetin (*P* > 0.05, Figure 2(c)).

#### 3.1.4. Quercetin Inhibited the Abnormal GSV Intima Thickening

**Figure 3:**
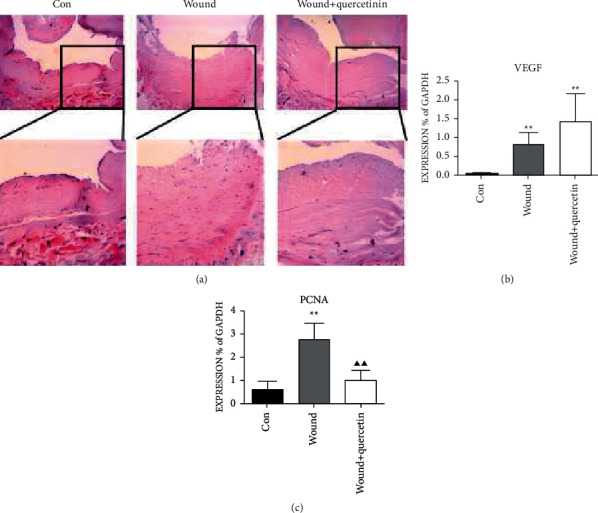
Quercetin inhibits the abnormal GSV intima thickening. (a) H&E staining of GSV showing the intima thickness (×200). The representative images for three groups are shown. The mRNA levels for (b) VEGF and (c) PCNA in the 3 groups of cultured GSV, which were pretreated by quercetin for 24 h. Statistical significance compared with H_2_O_2_ GSV vs. Con GSV is shown by an asterisk (^*∗*^*P* < 0.05, ^*∗∗*^*P* < 0.01) and with H_2_O_2_ GSV vs. H_2_O_2_ + quercetin GSV is shown by a trilateral (^*▲*^*P* < 0.05, ^*▲▲*^*P* < 0.01). Mean ± SD shown (*n* = 6).

**Figure 4:**
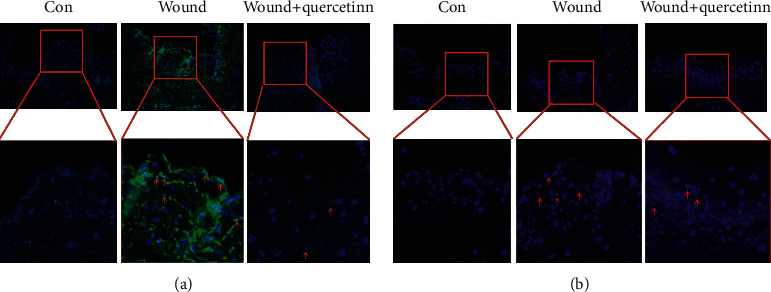
Quercetin inhibits the expression of PCNA and VEGF. (a) PCNA staining and (b) VEGF staining of GSV (×200). The representative images for three groups are shown. The positive cells are shown by arrowhead.

Mechanical damage often induces an abnormal vascular intima thickening. Therefore, we compared the GSV intima thickness by H&E staining (Figure 3(a)). As shown in figures, the intima of GSV in wound + quercetin in the group was thinner than that in the wound group. To explore the underlying mechanisms, we detected the expression of proliferation markers, PCNA and VEGF (Figures 3(b)-3(c)). The qPCR results revealed the expression of VEGF and PCNA was sharply increased after injury (*P* < 0.01), and quercetin could not influence VEGF transcription (*P* > 0.05), but suppresses the expression of PCNA. These results were consistent with immunofluorescence staining (Figure 4).

### 3.2. Discussion

Atherosclerosis is characterized by the formation of plaque in medium and large artery vascular wall, leading to tissue damage such as vascular obstruction or myocardial infarction. Endothelial dysfunction or activation is the first step in the pathogenesis of atherosclerosis. Cardiovascular disease risk factors lead to oxidative stress and oxidation of low-density lipoprotein (LDL) cholesterol. The oxidized LDL (oxLDL) attacks the arterial intima, releasing phospholipids to activate endothelial cells, leading to endothelial dysfunction. The oxLDL induced damage by activating the NF-*κ*B pathway with toll-like receptors (TLRs), which promoted expression of vascular cell adhesion factor (VCAM1), intercellular adhesion factor (ICAM-1), proinflammatory factor, and monocyte chemotactic factor (MCP1), raised mononuclear cells and caused endothelial damage, and accelerated atherosclerotic progression. During CABG progress, it is inevitable to cause GSV endothelial injury, which may accelerate the oxLDL accumulation. Many clinical and animal experiments have reported quercetin played good anti-inflammatory and antioxidant function [14, 15]. Reports have pointed out that quercetin itself has biological activities against cardiovascular disease and related risk factors [16]. Clinically, GSV is widely used as bridge graft in CABG surgery to supply blood to the heart. Mechanical damage and oxidation reaction are the key factors influencing the quality of GSV during CABG surgery. In this study, we explored the potential of quercetin in the protection of excised GSVs.

Increasing the intake of quercetin is beneficial for patients with cardiovascular disease. Duarte confirmed that increasing the quercetin-rich diet played an antihypertensive and antioxidant role in a spontaneously hypertensive rats model [17]. In addition to inhibiting thrombosis, quercetin also can serve as a vasodilator factor [18]. Studies about the vascular function have suggested that polyphenols can improve blood vessel function. The polyphenols in red wine can increase the biological activity of NO and improve arterial expansion [19]. Quercetin has a similar effect, improving vasodilatation by increasing the level of intracellular calcium ions [20]. Cogolludo put forward that quercetin improved blood vessel function by activating BKCa (large conductance Ca^2+^-activated K^+^ channels) [21]. These all showed that quercetin can protect blood vessels in vivo.

Current research studies on quercetin mainly focus on the protection of the venous endothelial cells. Although previous studies had showed that quercetin inhibited the generation of NADPH oxidase subunit p47 phox and NO, the concentration of quercetin in the experiments exceeded the physical standard, which led to the differences between the experiments and clinical studies. Angiotensin II induced venous endothelial cell dysfunction by promoting the synthesis of O^2-^ in the cell, reducing the NO production, strengthening the expression of NADPH oxidase subunit, and activating the PKC pathway. Jones used a physiological concentration of quercetin to confirm that quercetin can alleviate Ang II-induced vein endothelial cell damage, which may be related to less p47 phox and O_2_ generation [22].

In this experiment, we found that quercetin reduced the generation of NO and weakened injury induced by H_2_O_2_. But at pretreatment for about 1 h, the effect is not obvious. Considering the short processing time, we prolonged the time and found that quercetin showed good antioxidant ability after 24 h of treatment. Then, we tested the effect of quercetin on SOD activity and MDA production. The SOD and MDA reflect the extent of oxidative stress. We found that quercetin could obviously increase the SOD activity and reduce the generation of the MDA after H_2_O_2_ stimulation, indicating that quercetin could significantly reduce the oxidative damage to GSV in vitro.

Other researchers proposed that protective role of quercetin is closely related to the migration of smooth muscle cells (SMC). During vascular healing after damage, the migration of SMCs was influenced by MMP2 and MMP9, which could be inhibited by quercetin [23]. Thus, quercetin may play a antioxidant role through various pathways. Our study just reminds that quercetin may reduce the oxidation product by promoting the SOD activity, and further studies are needed for finding out these signal pathways.

Besides the antioxidant effect, quercetin also plays a strong anti-inflammatory role. It can significantly reduce the levels of serum c-reactive protein [24]. Adding it to the diet of mice or plant extracts containing can reduce obesity and insulin resistance induced by the high-fat diet and reduce systemic inflammatory responses. Laura fed mice with 50 *µ*g quercetin or nion extracts containing equal amount of quercetin for nine weeks and found proinflammatory cytokines in the adipose tissue of mice decreased, and chronic inflammatory cytokines, such as Cd11b, Cd68, F4/80, and Mcp1, also decreased, and serum level of IL-6 significantly reduced [25].

Similarly, we found that quercetin can reduce TNF*α* and IL-6 expression. TNF*α* is involved in systemic inflammatory response and is mainly generated during the acute inflammatory period, as an early proinflammatory cytokine that induces the downstream inflammatory responses. In the vein, TNF*α* can activate multiple signaling pathways, including C-SRC and ICAM-1, to initiate inflammatory response. IL-6 can induce the activation of neutrophils and endothelial cells, which releases inflammatory cytokines that recruit white blood cells. Quercetin can reduce TNF*α* and IL-6, inhibiting inflammatory response at the beginning, suggesting a good venous protection potential. Our study suggested that quercetin plays the anti-inflammatory effect by blocking TNF*α* and IL-6-associated signal pathways. It is consistent with Zhang's research [26]. Next, we detected the expression of C-C chemokine CCL20 (chemokine (C-C motif) ligand 20), the another proinflammatory cytokine. The CCL20 is a chemokine expressed mainly in the blood vessels, lymphatic tissue, and lung, produced by cells related with inflammation and autoimmune response such as endothelial cells, neutrophils, natural killer cells, and so on. It is well established that CCL20 contributes to inflammatory cell recruitment [27]. Some researchers believed that CCL20 is a mediator highly sensitive to the inflammatory response, and it could influence the endothelial cell migration [28]. Elnabawi et al. pointed that CCL20 was a potential biomarker of inflammation and impaired vascular health [29]. Regrettably, we found that the expression of CCL20 did not change with or without quercetin, indicating quercetin had no significant effect on CCL20 and lymphocyte chemotaxis.

NF-*κ*B pathways may play a role in the anti-inflammatory function of quercetin. Indra ever pretreated HUVECs with 125 *µ*M quercetin and found that it obviously inhibited leptin-induced ERK1/2 phosphorylation and NF-*κ*B activation, thereby alleviating inflammatory responses [30]. But they did not explore TNF*α*-related signal pathways due to insufficient RNA samples. Later, other researchers confirmed quercetin could obviously inhibit TNF*α*-related NF-*κ*B activation [31] and ROS generation [32]. Lu et al. proposed a potential mechanism of quercetin on regulating TNF*α*, using a atherosclerotic mice model induced by the high-fructose diet [33]. The NF-*κ*B signaling pathway is closely related to transcription of many inflammatory genes, such as IL-1b, IL-18, and TNF*α*. Studies have shown that activation of PI3K/AKT plays an important role in the inflammatory response balance. It was confirmed the long-term pressure leads to myocardial injury involving NF-*κ*B pathways, which is regulated by PI3K/AKT [34]. Considering that there are some common characters between the vascular inflammation and atherogenesis, Lu believed PI3K/AKT-regulated NF-*κ*B approach may also be involved in the atherosclerotic process induced by the high-fructose diet [33]. These results showed that phosphorylation of IKKa and IkBa activated NF-*κ*B signaling pathways, thus promoting the expression of IL-1*β*, IL-18, TNF*α*, and IL-6, and quercetin could inhibit inflammatory response by blocking NF-*κ*B signaling pathways and reduce the expression of proinflammatory cytokines involved in the PI3K/AKT pathway. Our study confirmed the quercetin showed obvious protection for the excised GSVs, just as the protection for the endothelial cells or smooth muscle cells alone in vitro.

Besides the inflammatory reaction, mechanical damage often induces abnormal wound healing and vascular intima thickening, caused by the disordered endothelial cell proliferation. Clinically, GSVs are the widely used bridge vessels in coronary artery bypass graft, and during the operation, GSVs are inevitably mechanical damaged, such as being sheared and sutured; then, the intima thickening and the vascular lumen is gradually narrowed, which influences the quality and blood flow of GSVs. The quality and blood flow of GSVs are essential to the outcomes of surgery and patients. Then, we evaluated the vascular intima thickness with H&E staining and endothelial cells proliferation by detecting PCNA and VEGF. The results showed the quercetin may restrain the abnormal intimal thickening by suppressing the endothelial cells proliferation after the injury and revealed its potential applications in CABG for a superior quality and blood flow of GSVs. Though quercetin shows great potential, it still has a long way to go in clinical application.

Compared to animal and cellular experiments, our study used human GSVs samples to demonstrate the protection of quercetin for oxidation reaction and inflammatory responses. However, application of human tissue can more accurately reflect the existing pathophysiological state of excised GSVs.

## 4. Conclusion

In summary, we demonstrated that quercetin can inhibit oxidation reaction, reduce inflammatory responses caused by mechanical damage induced, and inhibit the abnormal intimal thickening. The mechanisms involved are those regulated by production of NO, SOD, MDA, and TNF*α* signaling and endothelial proliferation.

## Data Availability

The datasets used and/or analyzed during the current study are available from the corresponding author upon request.

## Supplementary Materials

Supplementary Materials Table S1. Primer sequences required for QPCR analysis.
