# Supplementary material for: Quercetin Relieves the Excised Great Saphenous Vein Oxidative Damage and Inflammatory Reaction
Source: Evid Based Complement Alternat Med. 2021 Dec 31;2021:6251559. doi: 10.1155/2021/6251559 (PMC8741364; doi:10.1155/2021/6251559)
Supplement: Supplementary Materials — Table S1. Primer sequences required for QPCR analysis. [file 6251559.f1.docx]

**Table S1 Primer sequences required for QPCR analysis**

| Gene | Upstream sequence | Downstream sequence |
| --- | --- | --- |
| GAPDH | 5'-TGCACCACCAACTGCTTAGC-3' | 5'-GGCATGGACTGTGGTCATGAG-3' |
| IL-6 | 5'-CAGAGCTGTGCAGATGAGTACA-3' | 5'- GATGAGTTGTCATGTCCTGCAG-3' |
| TNF-α | 5'-CAAGCCTGTAGCCCATGTTGTA-3' | 5'- ACCAGCTGGTTATCTCTCAGCT-3' |
| CCL20 | 5'-TGGCCAATGAAGGCTGTGA-3' | 5'-GCGCACACAGACAACTTTTTCT-3' |
| PCNA | 5'-TTAAATTGTCACAGACAAGTAATGTCG-3' | 5'-TGGCTTTTGTAAAGAAGTTCAGGTAC-3' |
| VEGF | 5'-AGGAGGAGGGCAGAATCATCA-3' | 5'-CTCGATTGGATGGCAGTAGCT-3' |
